# Clinical evaluation of the Roche Elecsys® CMV IgG Avidity assay

**DOI:** 10.1007/s10096-014-2080-4

**Published:** 2014-03-02

**Authors:** C. Vauloup-Fellous, T. Lazzarotto, M. G. Revello, L. Grangeot-Keros

**Affiliations:** 1 UMR996, Univ Paris-Sud, 92140 Clamart, France; 2 AP-HP, Service de Virologie, Hôpital Paul Brousse, Villejuif, France; 3 U.O. di Microbiologia, DIMES, Policlinico Universitario St. Orsola-Malpighi, University of Bologna, Bologna, Italy; 4 Fondazione IRCCS Policlinico San Matteo, SC Ostetricia e Ginecologia, Pavia, Italy; 5 INSERM U764, Université Paris-Sud, AP-HP, Service de Virologie, Hôpital Paul Brousse, Villejuif, France

## Abstract

Congenital cytomegalovirus (CMV) infection has potentially severe consequences in newborns. The testing of pregnant women for CMV-specific antibodies may be useful for the identification of women at risk of transmitting the infection to the fetus. The determination of CMV IgG avidity helps to establish the timing of infection as IgG avidity matures during the course of infection. This study examines the performance of the Elecsys® CMV IgG Avidity assay using preselected samples from patients at different phases of CMV infection. The Elecsys® CMV IgG Avidity assay was tested at three sites using sequential samples from patients with recent primary CMV infection, as well as single samples from patients with recent primary or past CMV infection. The Elecsys® assay discriminated well between early (low avidity) and late (high avidity) phases of infection in sequential serum samples. Overall, 98.8 % of low-avidity samples corresponded to infection onset <180 days before sampling and 77.8 % of all high-avidity results corresponded to infection onset >90 days before sampling. The assay’s sensitivity was 90–97 %, with specificity ranging from 89 to 100 %, depending on the consideration of gray-zone avidity values. Single samples from recent primary or past infection showed similar distributions of avidity results. The Elecsys® CMV IgG Avidity assay results are in agreement with preselected samples from patients with primary or past CMV infection, showing that the test is an adequate predictor of the phase of infection.

## Introduction

Congenital cytomegalovirus (CMV) infection has potentially severe consequences in the newborn, which include neurosensory hearing loss, mental retardation, impaired physical development, and, in some cases, neonatal death [1]. The highest risk of transmission of CMV from mother to fetus is associated with primary infection, and the risk of severe abnormalities is highest when primary maternal infection occurs in the periconceptional period or the first trimester of pregnancy [2–4]. This risk decreases throughout pregnancy, although the chance of CMV vertical transmission is greatest in the third trimester [3–5]. Maternal recurrent CMV infection (i.e., a reinfection with a new CMV strain or reactivation of a latent strain) can also result in congenital CMV [6]. However, only rarely do non-primary infections lead to severe clinical symptoms.

The clinical diagnosis of maternal CMV infection is difficult, as symptoms are non-specific or absent in many cases. Hence, serological testing for CMV-specific immunoglobulin (Ig) M and IgG is required. IgM antibody is produced in the early phase of infection, and then declines in the following months, whereas IgG antibody levels increase gradually but can persist for several decades. This means that a subject who tests positive for CMV IgG only has been infected in the past. The scenario is much more complicated, however, if a subject tests positive for both IgM and IgG; in such a case, it is impossible to discriminate primary infection from a non-primary infection on a single serum sample [7]. Measuring the overall binding strength or avidity of CMV-specific IgG can help to make this distinction: primary infection generally will show low-avidity IgG in the early phases of infection, gradually maturing to high-avidity IgG approximately 22–24 weeks after infection onset [8, 9].

Ideally, assays designed to measure CMV IgG avidity should detect virus-specific IgG of low avidity in serum samples taken early after the onset (i.e., in the first 2–3 months) and IgG of high avidity in samples taken 6 or more months after infection onset, with IgG of moderate avidity detectable in between. However, a recent study showed that several currently available CMV IgG avidity assays do not achieve these ideal performance characteristics [10]. The aim of this study was to evaluate the recently developed Elecsys® CMV IgG Avidity assay, which is unique in that it uses a mixture of viral recombinant proteins rather than whole CMV antigen.

## Materials and methods

This study was carried out between March and July 2010 at three centers in Europe: Pavia, Italy; Bologna, Italy; and Paris-Clamart, France. Serum samples previously characterized at each of the centers were reanalyzed using the Elecsys® CMV IgM, IgG, and IgG Avidity assays.

### Preselection of samples

Frozen sequential serum samples taken from 61 pregnant women during routine clinical practice were reanalyzed at the center in Pavia (*n* = 180 in total). In 52 (85 %) of these cases, the diagnosis had been made on the basis of IgG seroconversion in the presence (33 cases) or absence of clinical symptoms. The remaining nine cases (eight symptomatic) had been diagnosed by CMV IgM kinetics (ETI-CYTOK-M reverse assay; DiaSorin, Saluggia, Italy), CMV IgG avidity (in-house CMV IgG avidity ELISA [11]), and CMV DNAemia [12]. When possible, presumed dating of infection onset was based on the presence of clinical symptoms and/or abnormal laboratory findings. Otherwise, the date of onset was arbitrarily set between the last IgG-negative and first IgG-positive sample (median time lapse 42, range 10–78 days).

Single frozen serum samples from subjects with primary (*n* = 100) and past CMV infections (*n* = 50) were reanalyzed at the center in Bologna. Primary infection had been confirmed by clinical history as well as CMV IgM-positive and low CMV IgG avidity results, positive DNAemia, and/or demonstration of seroconversion for CMV-specific antibodies. Past infection had been determined by the absence of CMV IgM and high CMV IgG avidity results. Negative IgM results were confirmed with immunoblot testing [13]. In addition, 52 sequential serum samples from 16 patients with primary CMV infection were also included in the study. Seven out of 16 (44 %) patients with primary CMV infection had symptoms that could have been related to CMV infection in an immunocompetent subject (fever and/or an increase in hepatic transaminases). In this group, the onset of infection was based on the appearance of symptoms. In the remaining nine asymptomatic patients (56 %), the date of onset was estimated to be 20–30 days (range, 15–40 days) before the date of seroconversion (for IgM or IgG), as previously reported [9]. For preclassification, sera were tested using the Enzygnost® CMV IgM assay and Enzygnost CMV IgG assay (Siemens Healthcare Diagnostics, Deerfield, IL, USA) and an in-house immunoblot for the detection of CMV-specific IgM [13]. CMV IgG avidity was tested with the Radim® Cytomegalovirus IgG Avidity EIA WELL assay (Radim, Pomezia, Italy) [9].

The center in Paris-Clamart reanalyzed single frozen serum samples from pregnant women with primary (*n* = 25) and past CMV infection (*n* = 57), as well as 48 sequential frozen serum samples from 14 pregnant women with primary CMV infection. The sequential samples had been taken monthly during pregnancy and included first visit samples containing CMV IgM with or without low-avidity CMV IgG and respective follow-up samples. The onset of infection was estimated to be 15–31 days before the first IgG-positive result. Samples had been preclassified using the Liaison® CMV IgG and CMV IgM assays (DiaSorin, Saluggia, Italy). If CMV IgM and IgG were both present, IgG avidity measurement was performed. Low-avidity results obtained with the Liaison CMV IgG avidity assay (DiaSorin, Saluggia, Italy) were confirmed using an in-house ELISA and the VIDAS® CMV IgG Avidity assay (bioMérieux, Marcy-L’Étoile, France).

### Elecsys® CMV Avidity assay: principle, calculation, and interpretation of results

The avidity assay consists of two measurements run in parallel using separate aliquots of the sample. The first is a reference measurement using undiluted sample or sample diluted as described above. In brief, the sample is incubated with a mixture of biotin- or ruthenium-labeled CMV-specific recombinant antigens to form sandwich complexes. Streptavidin-coated paramagnetic microparticles are added, and complexes are attracted to the solid phase by streptavidin–biotin interactions and a magnetic field. Unbound reagents are washed out of the system and bound antibody is detected by measuring voltage-induced chemiluminescence from the ruthenium labels. The second measurement follows the same reaction principle, but is performed in the presence of Elecsys® DilCMVAv buffer [0.8 M guanidine chloride, CMV-specific antigen (recombinant, *E. coli*); MES buffer 50 mmol/L, pH 6.5; preservative], which disrupts the binding of low-avidity IgG to CMV.

Testing with the Elecsys® CMV IgG Avidity assay is only applicable in samples that report a positive result with the Elecsys® CMV IgG assay. If an IgG result >500 U/mL is obtained, the sample should be diluted 1:20 with Elecsys® Diluent Universal prior to avidity determination.

The reference and DilCMVAv-treated measurement results are used to manually calculate the ratio of high-avidity IgG present in the sample, as follows:

$$ \mathrm{Avidity}\;\left(\%\mathrm{Avi}\right)=\frac{\mathrm{DilCMVAv}-\mathrm{treated}\;\mathrm{measurement}\;\left(\mathrm{U}/\mathrm{mL}\right)}{\mathrm{Reference}\;\mathrm{measurement}\;\mathrm{U}/\mathrm{mL}}\times 100 $$

Samples reporting %Avi values <45.0 indicated low-avidity IgG and samples with %Avi ≥55.0 indicate high-avidity IgG. Samples reporting %Avi ranging between 45.0 and 54.9 fall into the gray-zone and cannot be used to distinguish between past and active primary CMV infection. [14]

### Data analyses

Results from the Elecsys® CMV IgG avidity assay in sequential samples from patients with primary CMV infection were analyzed with respect to the percentage of samples that contained low-avidity, gray-zone, or high-avidity IgG within three time ranges: <90 days, 90–180 days, and >180 days after the onset of infection/first bleed. Samples not tested due to insufficient sample volume and samples with negative or equivocal IgG results were excluded from the analysis. Single samples from patients with primary or past CMV infection from the Paris-Clamart and Bologna sites were presented as multidot plots to show the distribution of avidity results using the Elecsys® CMV IgG Avidity assay from each site and by phase of infection. The sensitivity of the Elecsys® CMV IgG Avidity assay was defined as the percentage of samples predefined as representing a primary CMV infection that gave low-avidity results, whereas the specificity was defined as the percentage of predefined samples from past CMV infection that gave high-avidity results.

## Results

### Sequential samples from patients with primary CMV infection

**Fig. 1:**
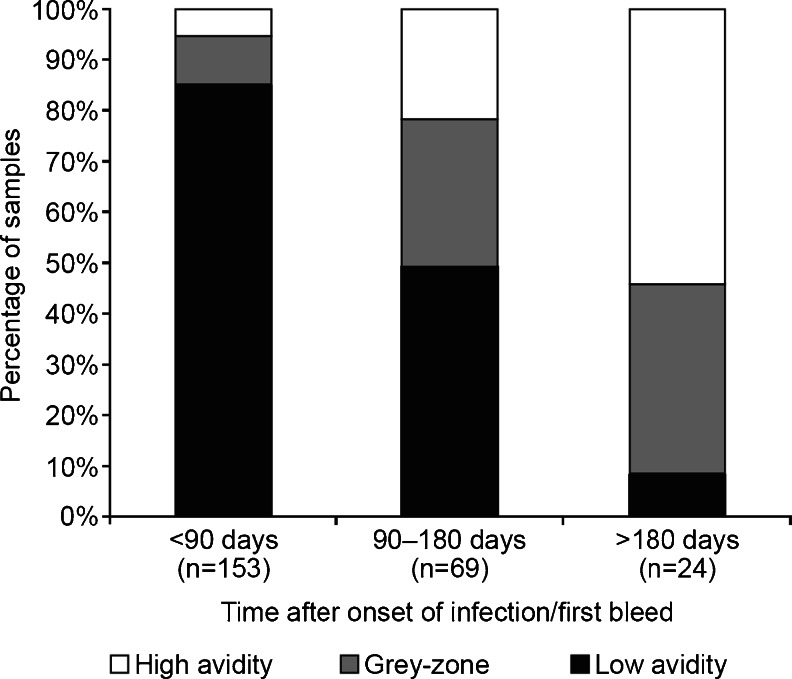
Distribution of CMV-specific IgG results from sequential samples at several time points after infection

In total, 246 sequential samples from patients with primary CMV infection from the three centers were included in the analysis. The distribution of CMV-specific IgG avidity results is shown in Fig. 1. Good discrimination between the early stages of infection (mainly low-avidity IgG results reported) and several months after infection (mainly high-avidity IgG results reported) was observed. There was some overlap in the distribution of the 90–180 days time period samples: 49 % showed low avidity, 29 % were equivocal, and 22 % showed high avidity. The majority of the low-avidity results corresponded with infection onset <90 days prior to the sample being taken, with only 1.2 % corresponding to infection onset >180 days before sampling. Samples containing high-avidity IgG corresponded to infection onset >90 days before sampling in 77.8 % of cases.

### Single samples from patients with primary or past CMV infection

**Fig. 2:**
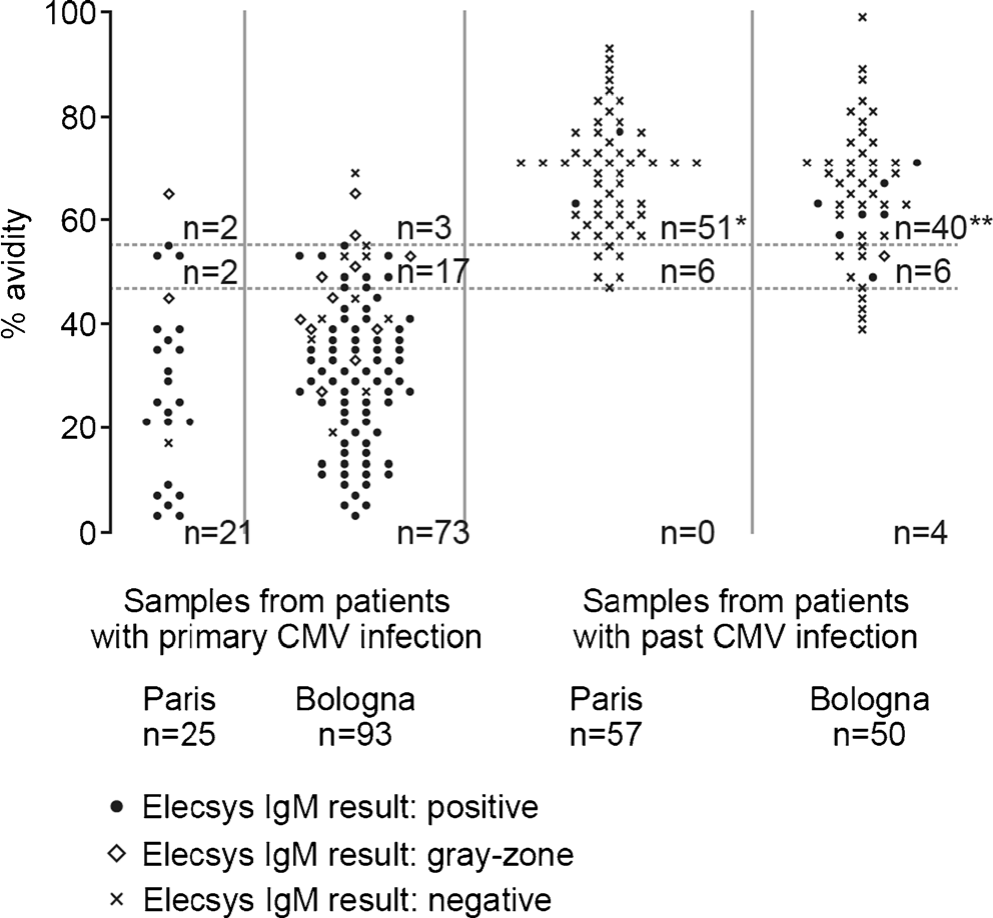
Distribution of CMV-specific IgG avidity results in single samples from patients with primary and past infections. *Three samples not shown due to avidity results >100 %. **Two samples not shown due to avidity results >100 %

The distribution of CMV-specific IgG avidity results in single samples from patients with primary or past CMV infection from the centers in Paris-Clamart and Bologna is shown in Fig. 2. The distribution of avidity results from the two centers in both primary and past infection samples was very similar. A clear distinction between the types of infection was observed, with primary infection samples showing low-avidity IgG results and past infection samples showing high-avidity IgG results. Discrepant results were relevant to high avidity obtained in five samples collected from different cases of primary infection, and four low-avidity results were observed in the past infection group of samples. Two of these four samples were retested with a third avidity assay and gave high-avidity results.

Assay sensitivity ranged from 91 to 97 % across the two testing centers (Table 1), and these values were very similar regardless of whether samples that fell into the gray-zone were excluded or were considered low-avidity samples. On the other hand, the specificity was slightly lower when samples with gray-zone avidity values were included in the low-avidity group (89.5 % in Paris-Clamart, 80.0 % in Bologna) than when they were excluded (100.0 % in Paris-Clamart, 90.9 % in Bologna; Table 2).

**Table 1:** Results from single samples from primary infection

	Paris-Clamart	Bologna
Samples tested	25	93^a^
Low avidity	21	74
Gray-zone	2	16
High avidity	2	3
Sensitivity^b^	91.30 %	96.10 %
95 % CI^b^	79.96–98.93	89.03–99.19
Sensitivity^c^	92.00 %	96.80 %
95 % CI^c^	73.97–99.02	90.86–99.33

^a^Seven samples excluded as they were IgG-negative with the Elecsys CMV IgG assay

^b^Results falling within the gray-zone were excluded from the analysis

^c^Results falling within the gray-zone were considered as low avidity for the analysis

**Table 2:** Results from single samples from past infection

	Paris-Clamart	Bologna
Samples tested	57	50
Low avidity	0	4
Gray-zone	6	6
High avidity	51	40
Specificity^a^	100.00 %	90.90 %
95 % CI^a^	93.02–100.00	78.33–97.47
Specificity^b^	89.50 %	80.00 %
95 % CI^b^	78.48–96.04	66.28–89.97

^a^Results falling within the gray-zone were excluded from the analysis

^b^Results falling within the gray-zone were considered as low avidity for the analysis

## Discussion

In this study, the performance of the Elecsys® CMV IgG Avidity assay was evaluated on two groups of previously characterized serum samples collected from subjects with primary or past CMV infection, respectively. The results appeared to correlate well with the sample classification.

In pregnancy, the most important requirement for a CMV IgG avidity assay is to help in identifying or excluding a primary infection occurring in the previous 3 months in women presenting with a positive CMV IgM result. To reflect this, the sequential samples from all three centers were divided into three time points: <90 days, 90–180 days, and >180 days after the onset of infection. The majority of samples taken <90 days after infection onset were reported as containing low-avidity CMV IgG and the majority of samples taken >180 days after infection onset were reported as high-avidity CMV IgG by the Elecsys® CMV IgG Avidity assay. A mixture of low-avidity, gray-zone, and high-avidity results (49 %, 29 %, and 22 % of samples, respectively) were observed in samples taken 90–180 days after infection onset. The latter observation is consistent with the fact that the kinetics of the IgG avidity maturation process is likely to vary between individuals. Because of this, low-avidity IgG can be detected later than 90 days after infection onset for some patients [8, 9], with a delay in IgG avidity maturation specifically observed in some pregnant women. This could explain why low-avidity results were reported for almost half of the samples taken 90–180 days after infection onset in this study. Differences in the method used to ascertain the date of infection onset at the three sites in this study may also contribute to this variation.

Using this assay, a primary infection would be identified with 95 % certainty, and a past infection would be identified with 92 % certainty. It should be noted that, in normal clinical practice, CMV IgG avidity measurements are only performed if patients also report a positive IgM result, so if the samples from this study taken >180 days after infection onset were the only samples that were tested for each of the patients, 15/24 samples would not actually have been assessed for CMV IgG avidity, as they were IgM negative.

The single samples from patients with primary or past CMV infection were analyzed separately because it is much more difficult to accurately date the presumed onset of infection when only single samples are available. Overall, the Elecsys® CMV IgG Avidity assay reported low-avidity IgG in ≈95 % of samples from primary infection and high-avidity IgG in ≈95 % of samples (≈85 % if equivocal results are considered low) from past infection.

Data from other studies indicate that the results from CMV IgG avidity assays vary greatly. Differences have been observed in the ability of assays to correctly detect or exclude recent infection [15, 16] and in the results reported by different assays for the same samples. A comprehensive study by Revello et al. [10] using eight commercial CMV IgG avidity assays to test 198 samples found that none of the results were the same for all assays. Furthermore, the same qualitative result obtained with at least five assays was only reported for 59.6 % of the samples, highlighting the lack of standardization between the assays and the need to test sequential follow-up samples with the same assay. In addition, delayed IgG avidity maturation has been observed in pregnant women with CMV [2, 8, 9, 15] or toxoplasma [17] infections, thus potentially leading to misclassification of the actual phase of infection. Finally, changing the cutoff level may be necessary in order to improve performance, as recently reported for the VIDAS assay [18].

In conclusion, the results obtained using the Elecsys® CMV IgG Avidity assay are in agreement with preselected samples classified as primary or past CMV infection. Therefore, the assay appears suitable for use in clinical practice.
